# Utility of a Gum-Elastic Bougie for Difficult Airway Management in Infants: A Simulation-Based Crossover Analysis

**DOI:** 10.1155/2015/617805

**Published:** 2015-10-01

**Authors:** Nobuyasu Komasawa, Akira Hyoda, Sayuri Matsunami, Nozomi Majima, Toshiaki Minami

**Affiliations:** Department of Anesthesiology, Osaka Medical College, 2-7 Daigaku-machi, Takatsuki, Osaka 569-8686, Japan

## Abstract

*Background.* Direct laryngoscopy with the Miller laryngoscope (Mil) for infant tracheal intubation is often difficult to use even for skilled professionals. We performed a simulation trial evaluating the utility of a tracheal tube introducer (gum-elastic bougie (GEB)) in a simulated, difficult infant airway model*. Methods.* Fifteen anesthesiologists performed tracheal intubation on an infant manikin at three different degrees of difficulty (normal [Cormack-Lehane grades (Cormack) 1-2], cervical stabilization [Cormack 2-3], and anteflexion [Cormack 3-4]) with or without a GEB, intubation success rate, and intubation time.* Results.* In the normal and cervical stabilization trials, all intubation attempts were successful regardless of whether or not the GEB was used. In contrast, only one participant succeeded in tracheal intubation without the GEB in the anteflexion trial; the success rate significantly improved with the GEB (*P* = 0.005). Intubation time did not significantly change under the normal trial with or without the GEB (without, 12.7 ± 3.8 seconds; with, 13.4 ± 3.6 seconds) but was significantly shorter in the cervical stabilization and anteflexion trials with the GEB.* Conclusion.* GEB use shortened the intubation time and improved the success rate of difficult infant tracheal intubation by anesthesiologists in simulations.

## 1. Background

Difficulty with airway management in pediatric patients is a major reason for cardiac arrest, brain injury, and death [1, 2]. Fewer studies have been conducted regarding difficult pediatric airway management, particularly in infants, compared to adult studies. Although several videolaryngoscopes and supraglottic devices have been developed for infants [3, 4], direct laryngoscopy with the Miller laryngoscope (Mil) remains the most widely used technique for infant tracheal intubation. However, tracheal intubation with Mil is sometimes difficult due to its suboptimal laryngoscopy view [5].

The gum-elastic bougie (GEB), a tracheal tube introducer, is commonly used in airway management and its use is recommended by national guidelines at early stages of difficult intubation [6, 7]. Various studies have been published regarding the utility of the GEB for difficult adult airway management, particularly for addressing difficult laryngoscopy situations [8, 9].

Clinical reports and evaluations of infant-size GEBs have yet to be fully validated. Thus, in this study, we compared the utility of the GEB for use by experienced anesthesiologists. As direct clinical evaluations are unethical, we performed validations with manikins. We hypothesized that the GEB would improve intubation in simulated difficult infant airways. To this end, we evaluated the utility of the GEB with respect to ease of tracheal intubation by experienced anesthesiologists on an infant manikin.

## 2. Materials and Methods

This study was approved by the Research Ethics Committee of Osaka Medical College (number 1321). As the study was not performed on human subjects, the clinical trial registration was not required. In July 2014, we selected 15 anesthesiologists with more than 5 years of clinical experience (12.2 ± 4.0 years) who received simulation training at Osaka Medical College. Written informed consent was obtained prior to the study.

**Figure 1:**
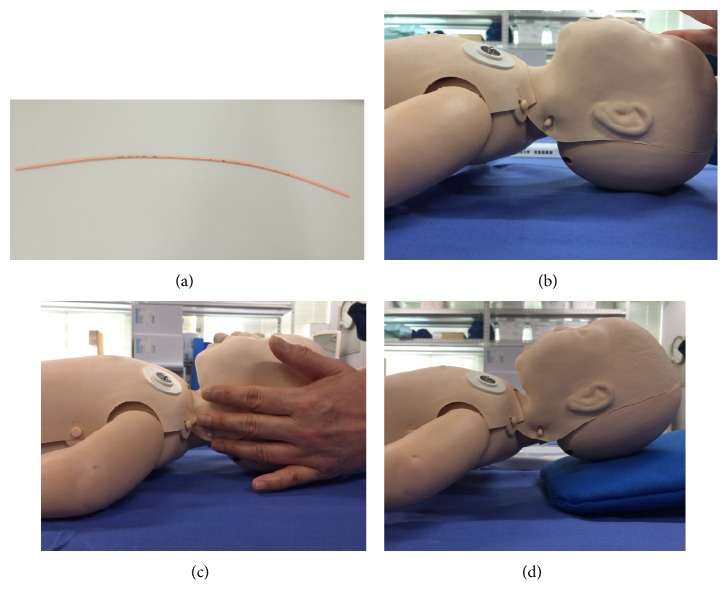
Gum-elastic bougie and manikins used in the study. (a) 5 Fr gum-elastic bougie, (b) ALS Baby Trainer manikin for the normal trial, (c) ALS Baby Trainer manikin for the cervical stabilization trial, and (d) ALS Baby Trainer manikin for the anteflexion trial.

The ALS Baby Trainer manikin (Laerdal, Stavanger, Norway), designed to accurately represent a three-month-old infant (weight: 11 pounds), was used in this study to simulate tracheal intubation. Intubations were performed using a Mil with a size 1 blade and a tracheal tube (Portex, St. Paul, MN, USA) with an internal diameter of 3.0 mm without a cuff. A 5 Fr tracheal tube introducer (Portex, St. Paul, MN, USA) was used as GEB because there is no infant-size GEB with angle tip commercially available (Figure 1(a)).

The manikin was placed on a flat and hard table and fixed to the table firmly with cohesive tape to prevent movement during laryngoscopy. To simulate difficult laryngoscopy, three trials were designed: normal trial (Cormack-Lehane grades (Cormack) 1-2), manual cervical stabilization (grades 2-3), and anteflexion (approximately 15 degrees; grades 3-4) (Figures 1(b)–1(d)).

The instructor explained to each participant how to intubate the tracheal tube with or without the GEB, attach a ventilation bag, and ventilate the lungs of the manikin. Although all participants had clinical experience with GEB usage, they were given five minutes to practice insertion, with the instructor available to give advice [10].

Participants were instructed to perform tracheal intubation within 60 seconds for each trial. We measured the insertion time from the start-point, that is, when participants took the device in their hands, to the end-point, that is, when they performed ventilation with a respiratory bag following insertion. Ventilation success or failure was determined by a visible chest rise and was judged by the same observer. Intubation times were recorded for both tracheal and esophageal intubations. In cases where participants could not intubate the traceha within 60 seconds, the trial was considered a failure and the intubation time was recorded as 60 seconds.

At the end of the trials, participants rated the difficulty of each trial on a visual analog scale (VAS) from 0 mm (extremely easy) to 100 mm (extremely difficult) for laryngoscopic imaging (VAS-LI) and passage of the tracheal tube through the glottis (VAS-PT) [11].

Results obtained from each trial were compared using two-way repeated measures analysis of variance for intubation time, and the chi-square test for successful ventilation. Data are presented as mean ± SD. *P* < 0.05 was considered statistically significant. This study used a randomized crossover design to minimize the learning-curve effect. The order of intervention was randomized for each participant by a random number table, resulting in a total of six interventions per participant.

Results of a nine-doctor preliminary study showed that the time required for successful intubation in the cervical stabilization trial was approximately 10 ± 4 s. We considered 5 s to be a clinically meaningful difference. Thus, we estimated that 12 participants would be adequate for two independent groups using *α* = 0.05 and *β* = 0.2.

## 3. Results

### 3.1. Success of Tracheal Intubation

Table 1 shows the number of successful intubations per trial. In the normal and cervical stabilization trials, all intubation attempts were successful with or without the GEB. In contrast, only one participant succeeded in tracheal intubation without the GEB in the anteflexion trial; success significantly improved with the GEB (*P* = 0.005).

### 3.2. Intubation Time

**Figure 2:**
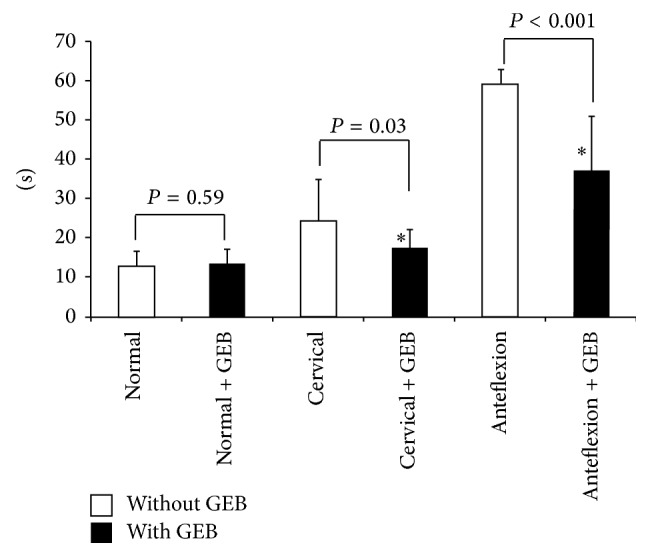
Intubation time with and without the gum-elastic bougie. White box: without GEB; black box: with GEB. Results are expressed as mean ± SD and were analyzed by two-way analysis of variance. NS: no significant difference; ^*∗*^ *P* < 0.05 compared to without GEB.

Results for intubation time are shown in Figure 2. Intubation times did not significantly differ with or without the GEB in the normal trial (without GEB, 12.7 ± 3.8 seconds; with GEB, 13.4 ± 3.6 seconds). Intubation time was significantly shorter with the GEB in both the cervical stabilization and anteflexion trials (cervical stabilization: without, 24.2 ± 10.6 seconds; with, 17.4 ± 4.7 seconds; *P* = 0.03; anteflexion: without, 59.0 ± 3.8 seconds; with, 37.2 ± 13.7 seconds; *P* < 0.001).

### 3.3. VAS for Laryngoscopic Imaging and Tube Passage through the Glottis

**Figure 3:**
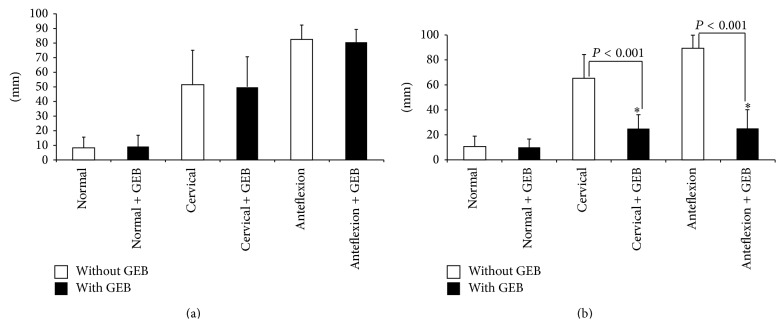
Visual analog scale for simulated tracheal intubation with and without gum-elastic bougie. (a) Laryngoscopic imaging and (b) tube passage through the glottis. White box: without GEB; black box: with GEB. Results are expressed as mean ± SD and were analyzed by two-way analysis of variance. NS: no significant difference; ^*∗*^ *P* < 0.05 compared to without GEB.

VAS-LI and VAS-PT scores are shown in Figure 3. VAS-LI scores did not significantly differ with or without the GEB in the three trials (Figure 3(a)). However, the VAS-LI score of the anteflexion trial was significantly higher than those of the cervical stabilization and normal trials. The VAS-LI score of the cervical stabilization trial was also higher than that of the normal trial.

The VAS-PT score did not significantly differ with or without the GEB in the normal trial. However, VAS-PT scores with the GEB were significantly lower than without GEB in the cervical stabilization and anteflexion trials (Figure 3(b)).

## 4. Discussion

Respiratory and airway problems are the most frequent causes of perioperative cardiac arrest in infants and children, highlighting the paramount importance of definite airway management [1, 12]. There have been only small evidences about pediatric difficult airway prediction factor, especially in infants [13]. In recent years, although many airway devices have been developed to address difficult intubation situations, most have been used in the context of adults and may not be suitable for pediatric use [14]. Hence, advances in adult airway management are not always transferable to pediatric practice. For example, most infants would not be able to tolerate awake or sedated intubation. Thus, options are limited for securing the infant airway.

Although the most widely used laryngoscope for these situations is the Mil, its difficulty to operate without experience can lead to an unacceptable high incidence of inaccurate intubation [15]. The GEB is a commonly used airway adjunct in intubation and is recommended by several guidelines for use at an early stage in cases of difficult intubation [6, 7]. Evidence from adult patients suggests that anesthesiologists can secure the airway with a high success rate when using the GEB [16].

Our present study demonstrated that intubation time is significantly shortened with the GEB under conditions of cervical stabilization, which simulates Cormack grades 2-3. Our findings suggest that GEB insertion is a simple and effective method for difficult infant airway management. However, in the anteflexion simulations with Cormack grades 3-4, about half of the participants failed in tracheal intubation. When anesthesiologists cannot see the GEB entering the larynx in Cormack grades 3-4, it is important to be able to determine whether the GEB is located in the trachea or esophagus. However, these signs in adults may not be applied to infant cases. Click and hold-up signs are well described in adults using GEB with angled tip. Click signs are apparent in adults and older children if GEB is correctly placed in the trachea [6, 17]. In this study, we used straight tip GEB. We should be careful that click sign can be confirmed in actual infants. Furthermore, hold-up sign will definitely work and is safe on manikin but there are concerns in real infant from the viewpoint of avoiding airway trauma. Thus, clinical evaluation of infant GEB including complications is needed in the future.

This study has several limitations. First, we used a manikin rather than real infants. Manikin simulation cannot mimic certain factors encountered in the clinical setting, such as body temperature, tissue stiffness, or sputum in the oropharynx. Second, though we utilized ALS Baby Trainer as infant simulator, more sophisticated or high-fidelity one such as SimBaby may provide more precise data. Third, we used 5 Fr tube exchanger because GEB for infant resembling the shape of adult one is not commercially available now. It is desirable that the GEB with angled tip will be developed. Fourth, the time required for airway intervention in a manikin is generally shorter than that required in actual infants [18]. Thus, accumulation of clinical data on GEB use in infant airway management is necessary.

At present, there are only case series suggesting the utility of GEB for Pierre Robin syndrome [17]. In the future, it will be important to accumulate more data or perform randomized controlled trial to validate whether the GEB is equally useful in difficult congenital airway situations [19]. Next, since the present study was conducted with experienced anesthesiologists, trials targeting novice doctors may further clarify the utility of the GEB for difficult infant airway management [20]. Assessing the utility of the GEB in emergent simulations, such as with chest compression, is also warranted. Several reports have been published regarding the utility of videolaryngoscopes for difficult infant airway management [3]. Though there are reports about utility of videolaryngoscope and GEB combination for difficult intubation in adult cases [21–23], there are no case reports on this combination in infant or pediatric cases. Clinical accumulation of these videolaryngoscopes when used in conjunction with the GEB may help developing new strategies for infant difficult airway management.

## 5. Conclusions

Our simulation study demonstrated that GEB use shortened intubation time and improved intubation success rate with difficult infant airways (e.g., cervical stabilization and anteflexion (Cormack 3-4)). However, in anteflexion trial, the success rate of tracheal intubation with the GEB was significantly lower than in cervical stabilization and normal airway trials.

## Figures and Tables

**Table 1:** Number of successful intubations.

	Without GEB (success/total)	With GEB (success/total)	*P* value
Normal (Cormack 1-2)	15/15	15/15	N.S.
Cervical stabilization (Cormack 2-3)	15/15	15/15	N.S.
Anteflexion (Cormack 4)	1/15	8/15	0.005

Successful intubations with or without the gum-elastic bougie (GEB) in the three trials. Differences were analyzed with the chi-square test.

## Notes

- The utility of tracheal tube introducers has previously been shown in adult cases, but not in pediatric cases, especially infants.
- GEB use shortened intubation time and improved the success rate of difficult infant tracheal intubation by anesthesiologists in simulations.
- GEB may be a useful device for difficult infant airway management.
